# Long-Term Outcomes in Patients with Dilated Left Ventricular Non-Compaction Versus Dilated Cardiomyopathy: A Propensity Score Matching Study

**DOI:** 10.3390/jcdd13090406

**Published:** 2026-08-23

**Authors:** Shiqi Gao, Ming Wu, Xiaoyu Ren, Shuyuan Zhang, Zeyuan Wang, Zhuang Tian, Shuyang Zhang

**Affiliations:** 1 Department of Internal Medicine, Peking Union Medical College Hospital, Chinese Academy of Medical Science & Peking Union Medical College, Beijing 100730, China; gaoshiqi@pumch.cn (S.G.); wuming18@pumch.cn (M.W.);; 2 Department of Cardiology, Peking Union Medical College Hospital, Chinese Academy of Medical Science & Peking Union Medical College, Beijing 100730, China; 3 Medical Research Center, State Key Laboratory of Complex Severe and Rare Diseases, Peking Union Medical College Hospital, Chinese Academy of Medical Science & Peking Union Medical College, Beijing 100730, China

**Keywords:** left ventricular non-compaction, dilated cardiomyopathy, echocardiography, propensity score matching, prognosis

## Abstract

The long-term prognosis of left ventricular noncompaction (LVNC) compared to dilated cardiomyopathy (DCM) remains inconclusive, as prior studies have frequently failed to adequately adjust for heart failure severity. We conducted a single-center, retrospective, comparative study enrolling 96 patients with dilated LVNC (dLVNC) and 437 patients with DCM diagnosed at Peking Union Medical College Hospital between January 2010 and December 2022. dLVNC was defined as meeting the Jenni criteria, with left ventricular ejection fraction (LVEF) ≤45% and increased left ventricular end-diastolic diameter (LVEDD). A 1:1 propensity score matching (PSM) was performed, with matching variables including sex, age, history of heart failure (HF) hospitalization, arrhythmia, LVEF, and LVEDD, resulting in 96 matched pairs. The primary endpoint was major adverse cardiovascular events (MACEs), comprising cardiovascular death or heart transplantation, HF hospitalization or cardiac resynchronization therapy implantation, severe arrhythmias, and systemic embolism. After a median follow-up of 5.46 (2.14–8.62) years in the dLVNC group and 5.26 (2.47–8.50) years in the DCM group, the Kaplan–Meier survival curves between the groups were highly overlapping and revealed no significant differences in MACEs (log-rank *p* = 0.88), all-cause mortality (log-rank *p* = 0.84), and individual components of MACEs. After adjusting for heart failure severity, dLVNC and DCM exhibit no substantial difference in long-term prognosis, and the morphological feature of excessive trabeculation itself does not independently increase the risk of adverse events.

## 1. Introduction

Left ventricular non-compaction (LVNC) is a myocardial morphological abnormality characterized by prominent trabeculations and deep intertrabecular recesses, with histological features showing significant thickening of the noncompacted myocardial layer and thinning of the compacted layer [1,2,3]. The widely accepted echocardiographic diagnostic criterion is the Jenni criteria, defined as a ratio of ≥2 between the thickness of the noncompacted and compacted layers at end-systole [1]. The prevalence of LVNC detected by echocardiography in the general population ranges from 0.02% to 0.14% [4,5], whereas it can reach up to 3% in patients with heart failure (HF) [6]. The clinical manifestations of LVNC are highly variable, ranging from complete asymptomatic status to progressive HF, malignant arrhythmias, and systemic thromboembolism, collectively known as the “LVNC triad.” Due to its heterogeneous manifestations, LVNC is frequently overlooked or misdiagnosed, making it one of the important causes of unexplained heart failure [7,8,9].

Regarding prognosis, LVNC has been classified as a primary cardiomyopathy that can follow a progressive course, with the potential to lead to significant morbidity and mortality. However, recent studies have consistently established that left ventricular systolic dysfunction is the core determinant of long-term outcomes in LVNC [10]. Early research revealed that adult LVNC-related mortality or heart transplantation occurred almost exclusively in patients with concurrent systolic dysfunction [8]. Systematic reviews and multiple large-scale cohort studies have further indicated that reduced left ventricular ejection fraction (LVEF) is the strongest independent risk factor for predicting major adverse cardiovascular events (MACEs), whereas LVNC patients without baseline systolic dysfunction generally exhibit a favorable prognosis [11,12,13,14]. Additionally, myocardial fibrosis characterized by late gadolinium enhancement (LGE) on cardiac magnetic resonance (CMR) imaging has been validated to further refine the risk stratification of LVNC [15,16]. These findings suggest that the prognostic stratification of LVNC depends to a large extent on the myocardial functional phenotype it presents, rather than the morphological trait of excessive trabeculation itself.

Based on accumulating evidence, the 2023 European Society of Cardiology (ESC) Guidelines for the Management of Cardiomyopathies marked a paradigm shift, explicitly discarding the classification framework that defined LVNC as a discrete cardiomyopathy based solely on morphological abnormalities [17]. The guidelines proposed a phenotype-oriented classification system, which categorizes cardiomyopathies into dilated cardiomyopathy (DCM), non-dilated left ventricular cardiomyopathy, hypertrophic cardiomyopathy, and other subtypes. LVNC, or left ventricular hypertrabeculation, is now characterized as a morphological trait that may manifest across multiple cardiomyopathy phenotypes, rather than being classified as an independent cardiomyopathy entity. Currently, the academic community is also increasingly inclined to abolish the term “LVNC cardiomyopathy” [18,19]. Under this new framework, when LVNC patients concurrently present with left ventricular dilation and severe systolic dysfunction, their phenotype fully aligns with the clinical definition of DCM and may be termed “dilated LVNC (dLVNC)”. However, a critical question emerges: does dLVNC, which exhibits a DCM phenotype, differ fundamentally in long-term prognosis from patients with classic DCM, or does the non-compaction morphological trait independently confer an additional risk of adverse events?

Currently, comparative studies addressing this question remain limited. A CMR study conducted in a DCM cohort demonstrated that excessive trabeculation in DCM does not confer independent prognostic value for predicting adverse cardiovascular events, including cardiovascular mortality, heart transplantation, left ventricular assist device implantation, cardiac arrest, or implantable cardioverter–defibrillator (ICD) shocks [20]. Similarly, a separate meta-analysis indicated that left ventricular noncompaction (LVNC) and DCM share a comparable overall cardiovascular risk profile; notably, LVNC may be associated with a higher risk of heart failure hospitalization, whereas no significant difference was observed in the risk of thromboembolic events between the two groups [10]. Underpinning this controversy is the core limitation of insufficient adjustment for the severity of heart failure in existing studies. Therefore, a study designed based on guideline-endorsed phenotypic concepts, with rigorous matching of cardiac functional status to compare long-term outcomes between dLVNC and classic DCM, would hold substantial clinical value. Such an investigation would clarify the independent clinical significance of excessive trabeculation, optimize risk stratification for these patients, and facilitate the implementation of phenotype-guided standardized pharmacotherapy.

## 2. Materials and Methods

### 2.1. Study Design and Population

**Figure 1:**
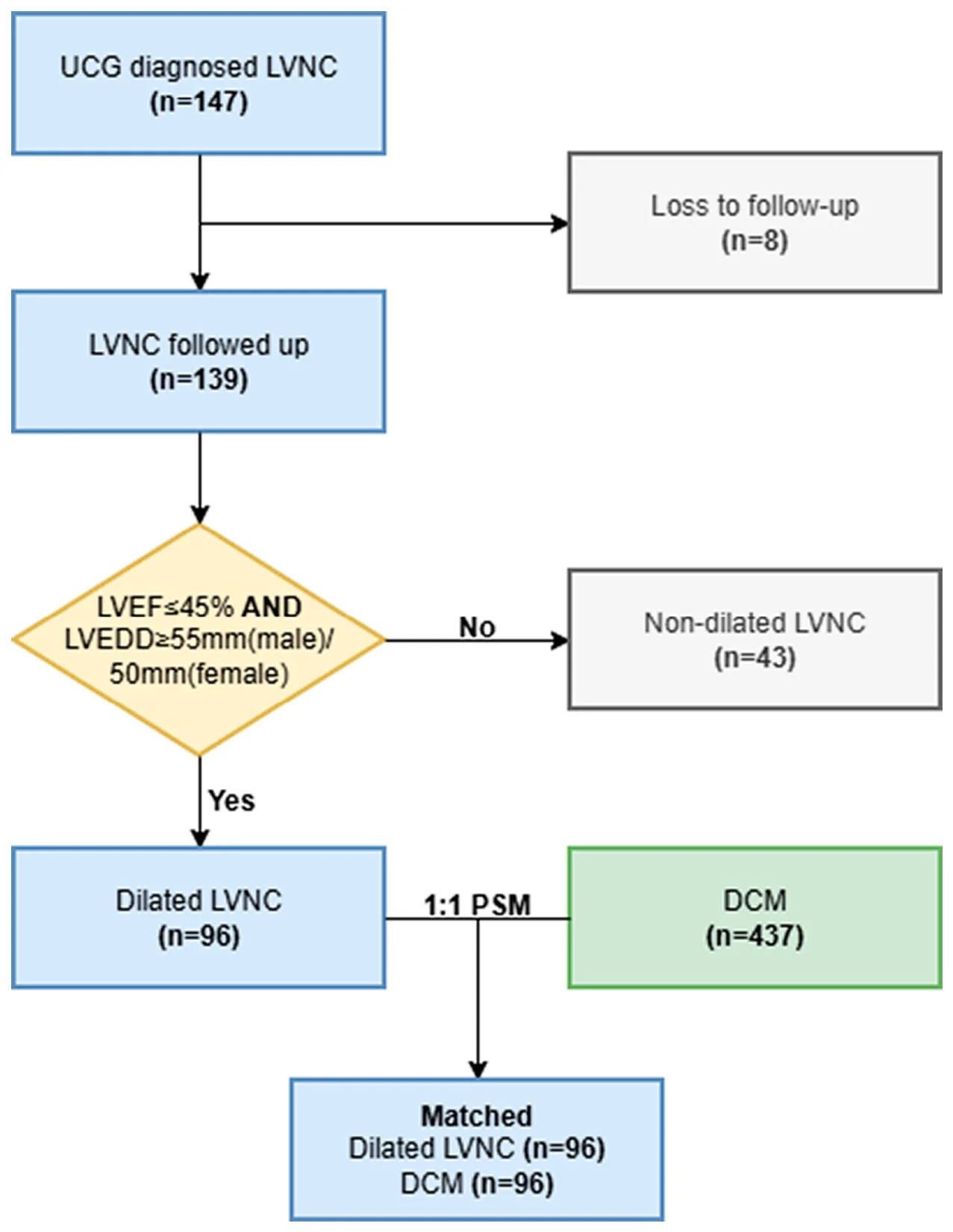
Study flowchart. The diagram describes the enrollment and propensity score matching flowchart of dilated LVNC and DCM patients. The dilated LVNC and DCM patients were 1:1 matched by age, sex, hospitalization for heart failure, arrhythmia, LVEF, and LVEDD. Abbreviations: UCG, echocardiography; LVNC, left ventricular non-compaction; LVEF, left ventricular ejection fraction; LVEDD, left ventricular end-diastolic diameter; DCM, dilated cardiomyopathy.

The flowchart for patient enrollment is presented in Figure 1. We conducted a single-center, observational, retrospective comparative study involving patients diagnosed with LVNC and dilated cardiomyopathy (DCM) at Peking Union Medical College Hospital (PUMCH) between 1 January 2010 and 31 December 2022. Patients’ diagnoses and clinical data were retrieved from the electronic medical record system. The Jenni criteria for echocardiography were adopted as the diagnostic standard for LVNC, defined as a ratio of noncompacted to compacted myocardial layer thickness (NC/C) ≥ 2 in any segment of the left ventricular short-axis view at end-systole [2].

Inclusion criteria were as follows: (1) diagnosis of LVNC confirmed by echocardiography at PUMCH; (2) age at diagnosis ≥ 14 years. Exclusion criteria included (1) coronary artery disease with ≥70% stenosis in one or more major coronary arteries, confirmed by coronary angiography or coronary computed tomography angiography; (2) hypertensive heart disease; (3) valvular heart disease causing significant hemodynamic abnormalities; (4) congenital heart disease with bidirectional or right-to-left shunts; (5) baseline echocardiographic data missing; and (6) prior diagnosis of malignant tumor within 5 years before LVNC diagnosis. LVNC diagnosis by echocardiography was independently assessed by two experienced cardiologists, and discrepancies were resolved in consensus during a joint evaluation with a third cardiologist.

Initially, 147 patients with LVNC were enrolled and followed up regularly via telephone or in-hospital visits. All follow-up procedures were completed by 31 December 2023, with 8 patients lost to follow-up. Among the 139 patients with successful follow-up, 96 were classified as dLVNC and 43 as non-dilated LVNC based on baseline LVEF and left ventricular end-diastolic diameter (LVEDD). The definition of dLVNC required fulfillment of the Jenni criteria, LVEF ≤ 45%, and LVEDD ≥ 55 mm (males) or ≥50 mm (females).

A control cohort of 485 DCM patients diagnosed during the same period at PUMCH was established. For DCM, the definitions of LVEF and LVEDD were identical to those for dLVNC, but with a maximum end-systolic NC/C ratio < 2; all other inclusion and exclusion criteria were consistent with those for LVNC. Follow-up for DCM patients also concluded on 31 December 2023, with 48 patients lost to follow-up.

Subsequently, 1:1 propensity score matching (PSM) was performed between the 96 dLVNC patients and 437 DCM patients, with matching variables including sex, age, history of HF hospitalization, arrhythmia, LVEF, and LVEDD. This yielded a matched cohort of 96 DCM patients with baseline characteristics comparable to those of the dLVNC group for subsequent analyses.

### 2.2. Variables

The variables included in this study encompassed demographic data, clinical characteristics, laboratory findings, electrocardiographic (ECG) parameters, echocardiographic metrics, LGE, and baseline therapeutic regimens. Basic demographic and clinical variables included sex, age, body mass index (BMI), smoking and alcohol consumption history, New York Heart Association (NYHA) functional class, history of heart failure hospitalization, and arrhythmias, as well as major comorbidities such as hypertension, diabetes mellitus, coronary artery disease (defined as a stenosis between 50% and 69% in any major coronary vessel on coronary angiography or coronary computed tomography angiography), dyslipidemia, and renal insufficiency (defined as estimated glomerular filtration rate [eGFR] < 60 mL/min/1.73 m^2^). Laboratory parameters included hemoglobin, alanine aminotransferase, serum creatinine, lipid profile, cardiac troponin I (cTnI), and N-terminal pro-B-type natriuretic peptide (NT-proBNP). Elevated cTnI was defined as exceeding the 99th percentile of the upper reference limit, while elevated NT-proBNP was defined as >125 ng/L. Key ECG features included QRS abnormalities, ST-T segment changes, left bundle branch block (LBBB), and ventricular arrhythmias. An abnormal ECG was defined as the presence of any of the following: non-sinus rhythm, abnormal QRS morphology, or repolarization abnormalities. Ventricular arrhythmias were defined as ≥2 ventricular beats per 10 s on a 12-lead ECG or ≥3 consecutive ventricular beats or ≥10,000 ventricular beats per 24 h on ambulatory Holter monitoring. Quantitative echocardiographic parameters included LVEDD, left ventricular end-systolic diameter, left ventricular shortening fraction, LVEF, interventricular septal thickness (IVS), left ventricular posterior wall thickness (LVPW), right ventricular anteroposterior diameter (RVD), and left atrial anteroposterior diameter (LAD). Qualitative echocardiographic parameters included left atrial (LA) enlargement, moderate or severe left ventricular diastolic dysfunction, and moderate or severe mitral regurgitation. Left atrial enlargement was defined as any one of the anteroposterior, transverse, or vertical diameters of the LA exceeding the upper limit of the reference range for the corresponding population. Enhanced CMR was performed in 58 dLVNC patients and 36 DCM patients, and CMR-derived LGE data were included only for subgroup analyses. The medications included pharmacological therapy and implantable devices at baseline.

### 2.3. Outcomes

The primary composite endpoint of the study was defined as MACE, comprising four sub-components: (1) cardiovascular death and heart transplantation; (2) hospitalization for HF or cardiac resynchronization therapy (CRT) implantation; (3) serious arrhythmic events, including ventricular fibrillation, sustained ventricular tachycardia, and appropriate ICD discharge; and (4) systemic embolic events, including ischemic stroke and peripheral arterial embolism with imaging confirmation. Secondary endpoints included all-cause mortality and a composite arrhythmic endpoint, defined as arrhythmic events specified in the primary endpoint, new-onset arrhythmias, and pacemaker or ICD implantation.

### 2.4. Statistical Analysis

In the present study, PSM was employed for patient matching, with a 1:1 ratio of dLVNC to DCM patients and a caliper of 0.02. Continuous variables are expressed as mean ± standard deviation or median (25th–75th percentiles), and normality was assessed using the Kolmogorov–Smirnov test. Categorical variables are presented as frequencies and percentages. Between-group comparisons of continuous variables were performed using Student’s *t*-tests (for normally distributed data) or Mann–Whitney U tests (for non-normally distributed data). For categorical variables, chi-square tests were used, with Fisher’s exact tests applied when appropriate. Kaplan–Meier survival curves were constructed to depict patient survival outcomes, and differences between curves were compared using the log-rank test. In subgroup analyses, hazard ratios (HRs) and their corresponding 95% confidence intervals (CIs) for the association between variables and outcomes were estimated via univariate Cox proportional hazards regression models. A two-tailed *p* value < 0.05 was considered statistically significant for all analyses. Statistical analyses and visualization of results were performed using IBM SPSS Statistics 25.0 (IBM Corp., Armonk, NY, USA) and R software version 4.2.1 (R Foundation for Statistical Computing, Vienna, Austria).

## 3. Results

### 3.1. Baseline Characteristics

**Figure 2:**
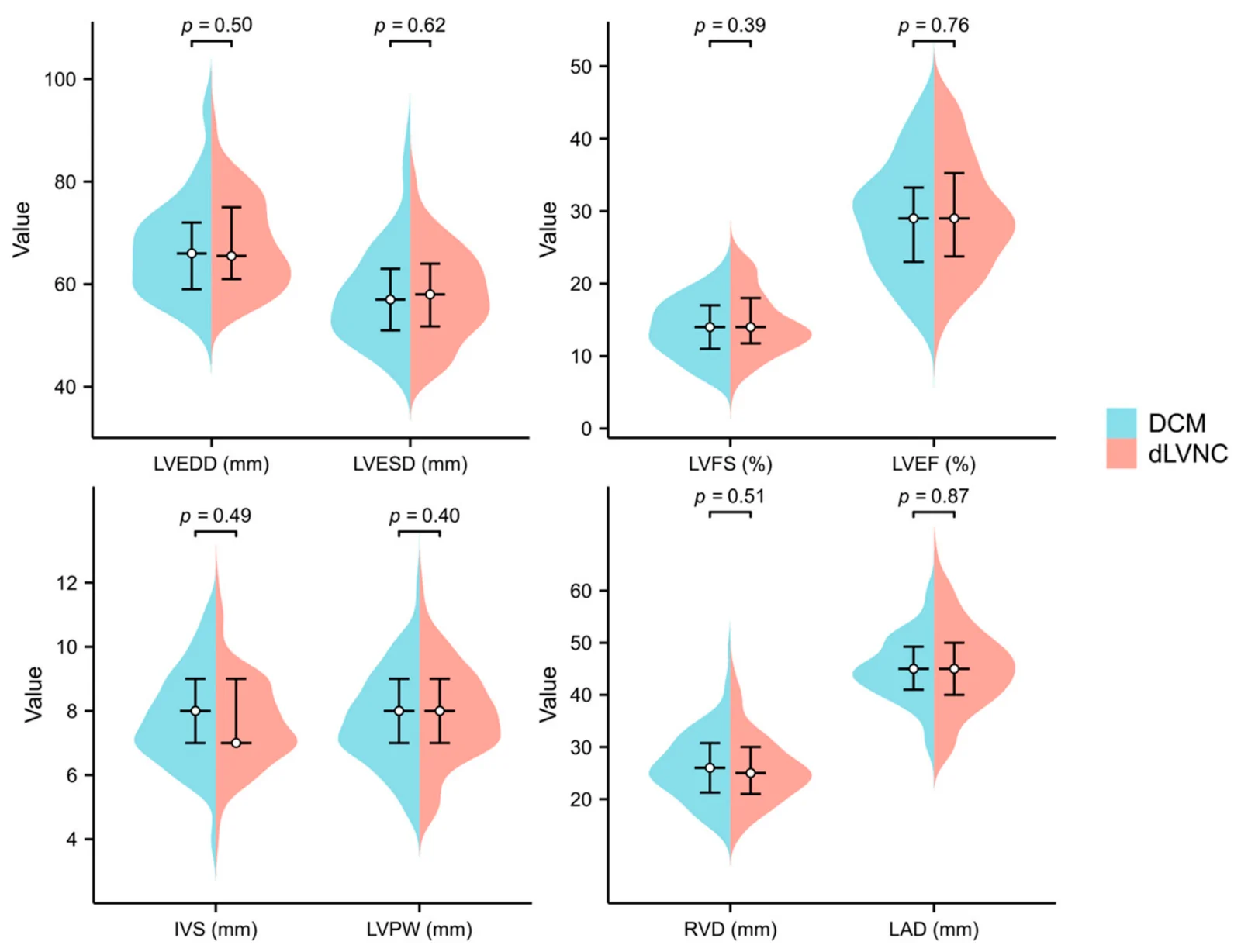
Echocardiographic parameters of dLVNC and DCM patients. The figure shows the distribution of quantitative echocardiographic parameters of dLVNC and DCM patients, including the median and quartiles. *p* values were calculated using the Mann–Whitney U test. Abbreviations: DCM, dilated cardiomyopathy; dLVNC, dilated left ventricular non-compaction; LVEDD, left ventricular end-diastolic diameter; LVESD, left ventricular end-systolic diameter; LVFS, left ventricular fraction of shortening; LVEF, left ventricular ejection fraction; IVS, interventricular septum; LVPW, left ventricular posterior wall; LAD, left atrial anteroposterior diameter; RVD, right ventricular anteroposterior diameter.

Baseline characteristics of 96 patients with dLVNC and 96 patients with DCM are summarized in Table 1. The mean age of the dLVNC group was 47.02 ± 16.43 years, compared with 44.07 ± 15.76 years in the DCM group (*p* = 0.21), and the proportion of male patients was 61.5% and 53.1%, respectively (*p* = 0.24). Most demographic and basic clinical characteristics, laboratory parameters, and electrocardiographic features were well-balanced and comparable between the two groups. Variables with significant intergroup differences included hypertension, BMI, and serum creatinine levels. Regarding echocardiographic parameters, no statistically significant differences were observed in cardiac structure and function between the two groups; the distributions of atrial and ventricular dimensions, wall thickness, and LVEF were highly consistent (Figure 2). The LVEF values were 29 (24–35)% in the dLVNC group and 29 (23–33)% in the DCM group (*p* = 0.76). Among patients who underwent CMR imaging, LGE was 65.5% positive in the dLVNC group and 66.7% positive in the DCM group (*p* = 0.91). The DCM group exhibited a higher baseline medication rate. Specifically, the proportions of patients receiving angiotensin-converting enzyme inhibitors/angiotensin receptor antagonist/angiotensin receptor neprilysin inhibitor (92.7% vs. 82.3%, *p* = 0.03) and mineralocorticoid receptor antagonists (91.7% vs. 51.9%, *p* = 0.001) were significantly higher in the DCM group than in the dLVNC group.

### 3.2. Clinical Outcomes

Clinical outcomes of the two groups are summarized in Table 2. The median follow-up duration was 5.46 (2.14–8.62) years in the dLVNC group and 5.26 (2.47–8.50) years in the DCM group. During follow-up, 59 patients in each group experienced MACEs, yielding an identical MACE incidence rate of 61.5% in both cohorts. All-cause mortality occurred in 17 patients (17.7%) in the dLVNC group and 18 patients (18.8%) in the DCM group. No statistically significant differences were observed between the two groups in the incidence of individual major sub-endpoints or secondary endpoints. Specifically, the DCM group exhibited slightly higher rates of cardiovascular death or heart transplantation, as well as hospitalization for HF or CRT implantation. Conversely, the dLVNC group had a higher number of systemic embolic events. However, none of these between-group differences reached statistical significance.

### 3.3. Survival Analyses

**Figure 3:**
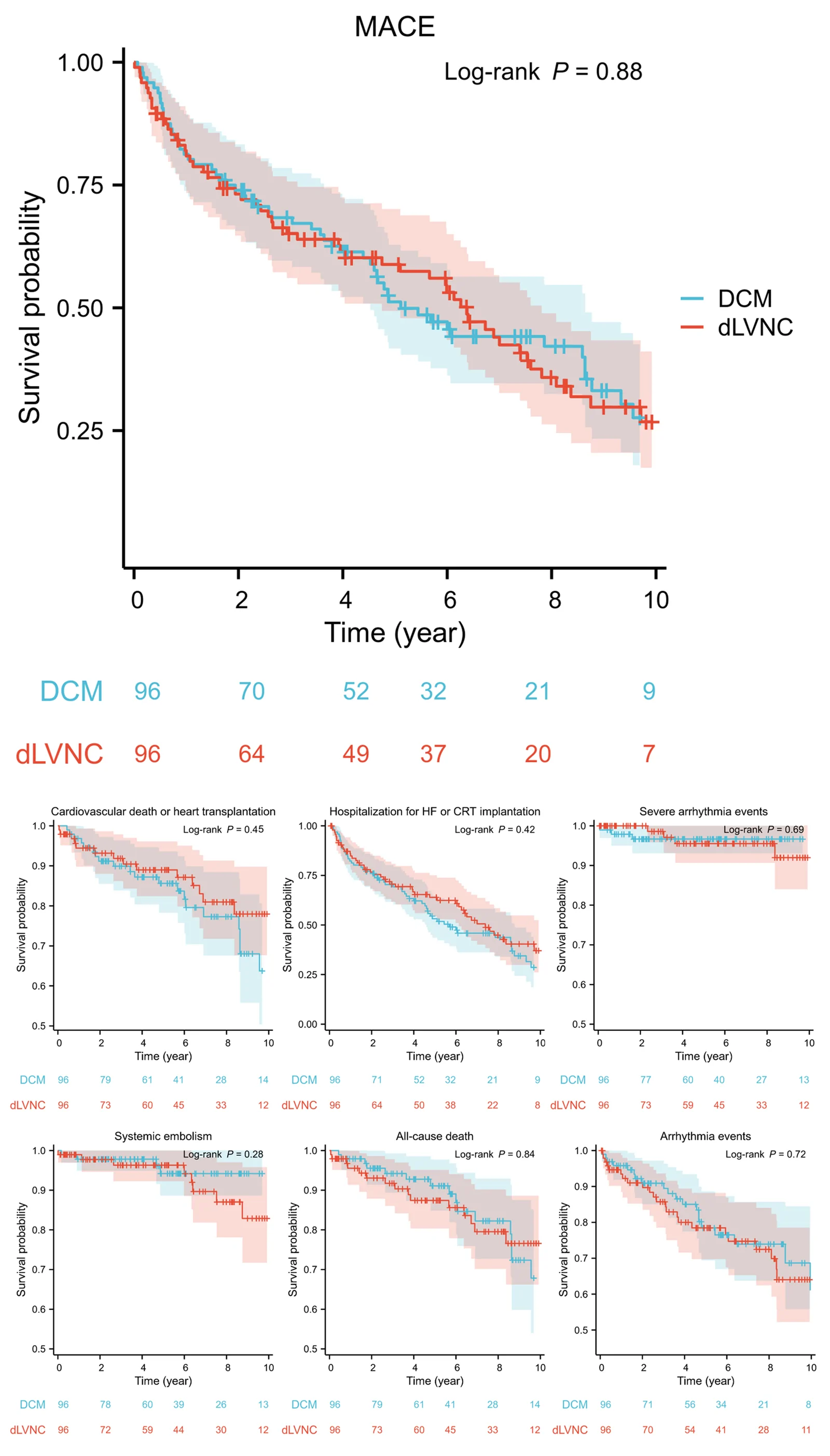
Comparison of outcomes between dLVNC and DCM patients. The figures show the Kaplan-Meier curves of dLVNC and DCM patients on MACE, cardiovascular death or heart transplantation, hospitalization for HF or CRT transplantation, severe arrhythmias, systemic embolisms, all-cause death and composite arrhythmias, respectively. The shadows represent the 95% confidence intervals of the survival curves. The *p* values were calculated by the log-rank test. The corresponding numbers at risk are presented below each curve. Abbreviations: DCM, dilated cardiomyopathy; dLVNC, dilated left ventricular non-compaction; MACE, major adverse cardiovascular event; HF, heart failure; CRT, cardiac resynchronization therapy.

The Kaplan–Meier survival curves for 96 dLVNC patients and 96 DCM patients across various clinical outcomes are shown in Figure 3. The survival curves of the two groups demonstrated a high degree of overlap in the primary endpoint, secondary endpoint, and individual components of MACEs. Log-rank tests revealed no significant differences in risk between the groups for MACEs (*p* = 0.88), cardiovascular death or heart transplantation (*p* = 0.45), hospitalization for HF or CRT implantation (*p* = 0.42), severe arrhythmia events (*p* = 0.69), systemic embolisms (*p* = 0.28), all-cause mortality (*p* = 0.84), or arrhythmic events (*p* = 0.72).

The figures show the Kaplan–Meier curves of dLVNC and DCM patients on MACEs, cardiovascular death or heart transplantation, hospitalization for HF or CRT transplantation, severe arrhythmias, systemic embolisms, all-cause death, and composite arrhythmias. The shadows represent the 95% confidence intervals of the survival curves. *p* values were calculated by the log-rank test. The corresponding numbers at risk are presented below each curve. Abbreviations: DCM, dilated cardiomyopathy; dLVNC, dilated left ventricular non-compaction; MACEs, major adverse cardiovascular events; HF, heart failure; CRT, cardiac resynchronization therapy.

### 3.4. Subgroup Analyses

**Figure 4:**
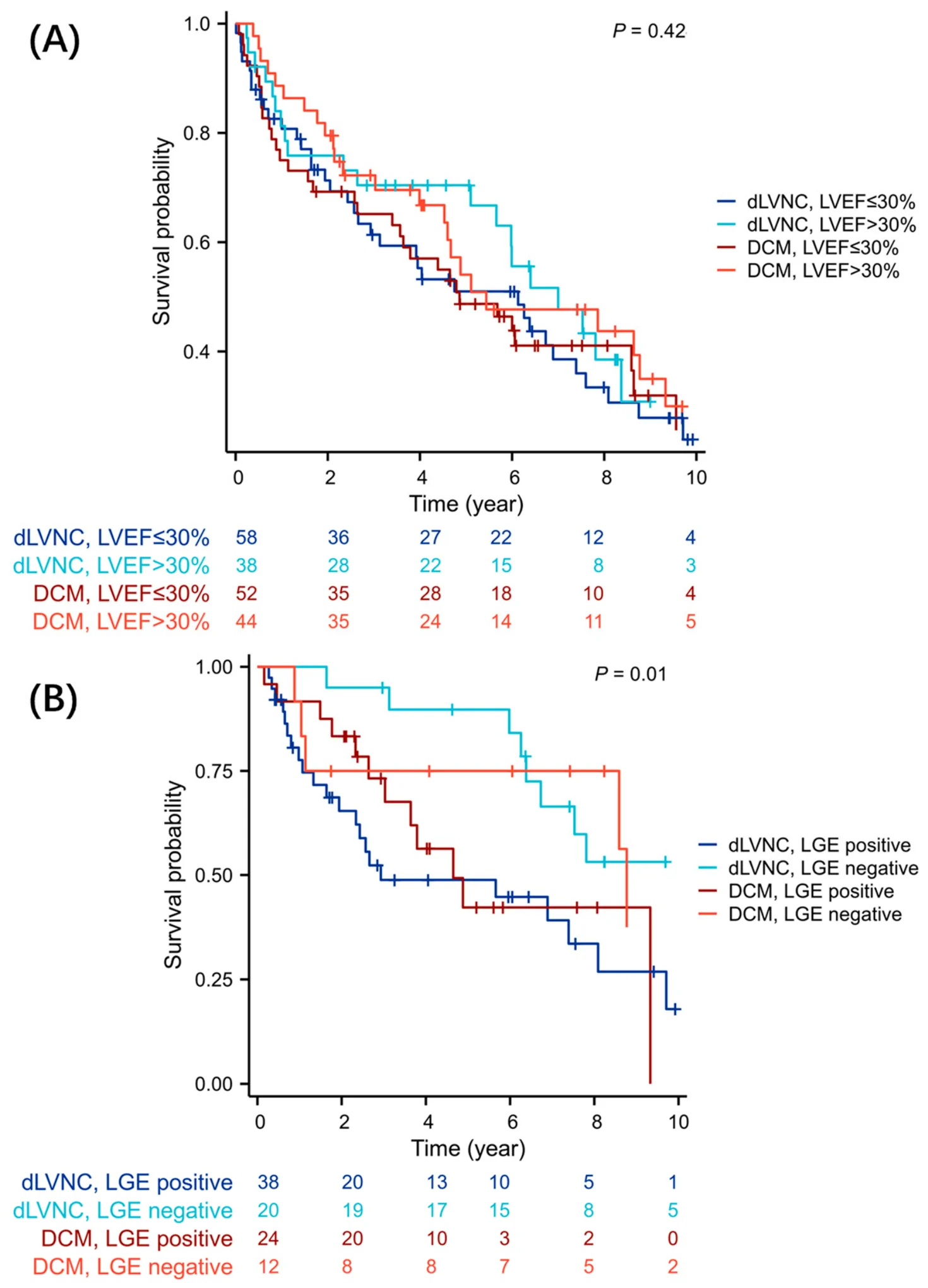
Comparison of MACEs between dLVNC and DCM patients stratified by LVEF and LGE. (**A**) shows the Kaplan-Meier curves of dLVNC and DCM patients on MACE, stratified by LVEF. (**B**) show the Kaplan-Meier curves of dLVNC and DCM patients on MACE, stratified by LGE. The *p* values were calculated by the log-rank test. The corresponding numbers at risk are presented below each curve. Abbreviations: DCM, dilated cardiomyopathy; dLVNC, dilated left ventricular non-compaction; MACE, major adverse cardiovascular events; LVEF, left ventricular ejection fraction; LGE, late gadolinium enhancement.

The results of univariate Cox regression for the association between LVEF ≤ 30% or positive LGE and MACEs are presented in Table 3. Specifically, LGE was associated with a significantly elevated MACE risk in dLVNC patients (HR 3.13; 95% CI 1.38–7.07; *p* = 0.006). In contrast, no significant association between LGE and MACE risk was observed in the DCM group (HR 1.93; 95% CI 0.64–5.83; *p* = 0.24), which was likely attributable to the limited sample size (only 36 patients underwent CMR). Notably, LVEF ≤ 30% did not significantly increase MACE risk in either the dLVNC (HR 1.34; 95% CI 0.78–2.32; *p* = 0.29) or DCM group (HR 1.39; 95% CI 0.82–2.34; *p* = 0.22). Survival curves stratified by LGE and LVEF are provided in Figure 4. The log-rank test indicated a statistically significant difference in survival outcomes among the four subgroups stratified by LGE status across dLVNC and DCM cohorts (log-rank *p* = 0.01). In contrast, no significant difference was detected among the four subgroups correspondingly stratified by LVEF (log-rank *p* = 0.42).

Figure 4A,B show the Kaplan–Meier curves of dLVNC and DCM patients on MACEs, stratified by LVEF and LGE, respectively. *p* values were calculated by the log-rank test. The corresponding numbers at risk are presented below each curve. Abbreviations: DCM, dilated cardiomyopathy; dLVNC, dilated left ventricular non-compaction; MACEs, major adverse cardiovascular events; LVEF, left ventricular ejection fraction; LGE, late gadolinium enhancement.

## 4. Discussion

To the best of our knowledge, this study represents the first long-term prognostic comparison between baseline-matched patients with DCM and LVNC. The core finding of the study is that following rigorous PSM, patients with dLVNC and DCM exhibited highly comparable long-term outcomes across hard endpoints, including MACEs and all-cause mortality, with nearly identical survival curves. The results provide critical insights for re-evaluating the disease classification and clinical management strategies of LVNC. Consistent with our conclusions, a prognostic analysis of CMR data from 162 DCM patients similarly demonstrated that the NC/C ratio or non-compacted myocardial mass fraction was not an independent prognostic factor for all-cause mortality in DCM patients [18]. By applying PSM to further strictly control confounding variables and expand the spectrum of endpoint events, our study still observed no significant differences in long-term prognosis between dLVNC and DCM, confirming that the feature of myocardial non-compaction does not impose additional clinical risk on DCM phenotype patients with ventricular dilation and systolic dysfunction.

Our findings also corroborate that the primary clinical risk of LVNC arises from reduced cardiac function from an alternative perspective and strongly support the phenotype-driven prognosis paradigm in cardiomyopathies. Previous studies on myocardial trabeculation in DCM often failed to adequately adjust for the severity of heart failure, serving as a critical confounding factor. However, the present study strategically focused on a dLVNC subgroup with established left ventricular enlargement and significantly reduced LVEF and utilized PSM to balance core prognostic factors, including age, sex, LVEF, LVEDD, and history of HF hospitalization. Under these conditions, the morphological feature of non-compaction did not independently increase the risk of cardiovascular events, including cardiovascular death, HF, and severe arrhythmias, suggesting that once LVNC progresses to the dilated phenotypic stage, the core drivers of patient prognosis are myocardial systolic dysfunction and ventricular remodeling, rather than the embryonic arrest of myocardial compaction itself. Therefore, dLVNC may be more appropriately classified as one of the multiple etiologies underlying the DCM phenotype, rather than a distinct disease entity with independent prognostic characteristics, which holds potential implications for disease taxonomy. The relatively low incidence of severe arrhythmias observed in this study may be associated with the high baseline rate of CRT therapy. Previous large-scale meta-analyses have demonstrated that CRT significantly reduces the occurrence of malignant ventricular arrhythmias, as evidenced by a reduction in appropriate ICD discharges [21], further supporting the role of CRT as primary prevention in suitable patients. On the other hand, while the incidence of systemic embolism events was numerically higher in the dLVNC group, no statistically significant difference was observed, which cannot fully exclude the issue of insufficient statistical power. Furthermore, a large-scale comparative study has confirmed a higher stroke risk associated with AF in LVNC patients [22], indicating that the traditional concern regarding thrombus formation within non-compacted trabeculae should still be maintained. However, the relatively high baseline rate of antiplatelet or anticoagulant use may have significantly reduced the incidence of stroke events during follow-up, making it difficult to compare stroke risk between the two groups under natural disease progression.

On the other hand, the subgroup analysis in this study provides a tool for precise risk stratification of patients. Numerous existing studies have already confirmed that LGE is an independent adverse prognostic factor for DCM [23,24]. In our study, LGE was associated with a 3.13-fold increased risk of MACEs in dLVNC patients, further validating the core prognostic value of myocardial fibrosis in LVNC; due to sample size limitations (36 DCM patients underwent CMR imaging), no significant association was found between LGE and MACE risk in the DCM group. In contrast, the study found that LVEF ≤30% did not exhibit a significant risk increment in both matched cohorts, which may be attributed to the sample size constraint and the risk-mitigating effect of modern pharmacotherapies on patients with low ejection fraction. This suggests that for dLVNC and DCM patients who have already developed reduced ejection fraction, risk assessment relying solely on LVEF values may lack sufficient sensitivity. As a direct biomarker of myocardial fibrosis, LGE can effectively identify high-risk patients and inform decisions regarding more intensive follow-up and implantable device interventions.

Our findings indicate that among patients with dLVNC, adverse events are driven by systolic dysfunction and remodeling, not by trabeculation itself. Therefore, a shift to function-based risk stratification is essential, as endorsed by the 2023 ESC guidelines, which reclassify LVNC as a trait rather than a distinct cardiomyopathy. For dLVNC patients, management should mirror DCM-focused protocols: prioritize LVEF monitoring and myocardial fibrosis assessment over repeated morphologic phenotyping. Adopting this function-first approach would reduce unnecessary interventions and align clinical practice with contemporary phenotype-driven frameworks.

This study has several limitations. The single-center retrospective design, limited sample size, and incomplete matching of baseline variables (e.g., hypertension and renal function) may introduce residual confounding. An imbalance in key therapeutic interventions, particularly baseline medications between the groups, also exists. Additionally, the long follow-up period required for monitoring long-term patient outcomes coincides with the emergence of novel therapies over the past decade, such as sodium-glucose cotransporter 2 inhibitors, which have further improved the prognosis of HF patients and may exert a left ventricular reverse remodeling effect in LVNC [25], potentially contributing to internal heterogeneity in the prognosis of the study population. Owing to the observational study design, only baseline medication status was included, thus precluding the incorporation of dynamic medication adjustments during follow-up. Furthermore, as LGE was assessed only as a binary variable and the cohort size was modest, the ability to detect the prognostic significance of fibrosis burden is limited. Future multicenter investigations incorporating quantitative LGE analysis are warranted to clarify whether the extent of myocardial scar offers additional risk stratification beyond LVEF and can guide patient selection for ICD therapy. Finally, genetic testing was not performed in this cohort, precluding analysis of genotype–phenotype correlations. Future studies incorporating systematic genetic screening are needed to determine whether specific variants provide additional prognostic information.

## 5. Conclusions

In summary, by balancing cardiac function status, this study robustly demonstrates that dLVNC and DCM share comparable long-term prognosis, which holds practical implications for clinical practice: for dLVNC patients, standardized HF management aligned with DCM guidelines should be fully implemented; efforts should be made to maximize the utilization of guideline-directed medical therapy to achieve target levels; and LGE should be actively employed for risk stratification, thereby effectively improving patients’ long-term outcomes.

## Figures and Tables

**Table 1:** Baseline characteristics of dLVNC and DCM patients.

Baseline Characteristics	dLVNC (*n* = 96)	DCM (*n* = 96)	*p* Value
Demographic and clinical characteristics			
Male	59 (61.5)	51 (53.1)	0.24
Age, years	47.0 ± 16.4	44.1 ± 15.8	0.21
NYHA cardiac function class			
I	9 (9.4)	10 (10.4)	0.77
II	44 (45.8)	38 (39.6)	
III	27 (28.1)	33 (34.4)	
IV	16 (16.7)	15 (15.6)	
Hospitalization for heart failure	74 (77.1)	79 (82.3)	0.37
Arrhythmia	73 (76.0)	70 (72.9)	0.62
Hypertension	21 (21.9)	36 (37.5)	0.02
Diabetes mellitus	10 (10.4)	18 (18.8)	0.10
Coronary heart disease	8 (8.3)	6 (6.3)	0.58
Dyslipidemia	37 (38.5)	29 (30.2)	0.18
Renal dysfunction	10 (10.4)	5 (5.2)	0.18
BMI, kg/m^2^	23.3 ± 3.9	24.8 ± 4.4	0.02
Cigarette consumption	29 (30.2)	19 (20.0)	0.10
Alcohol intake	18 (18.8)	11 (11.5)	0.16
Laboratory examinations			
HGB, g/L	141 ± 22	139 ± 21	0.58
ALT, U/L	27 (16–41)	28 (18–47)	0.56
Scr, μmol/L	80 (68–97)	77 (60–92)	0.04
FBG, mmol/L	5.2 (4.6–5.6)	5.3 (4.8–6.1)	0.07
TC, mmol/L	4.13 (3.37–4.76)	4.00 (3.27–4.78)	0.39
TG, mmol/L	1.20 (0.77–1.75)	1.25 (0.89–1.57)	0.80
HDL-C, mmol/L	1.01 (0.81–1.14)	0.92 (0.74–1.11)	0.12
LDL-C, mmol/L	2.61 (2.03–3.33)	2.49 (1.99–3.16)	0.37
cTnI, ng/L	30 (6–76)	40(0–91)	0.20
cTnI elevation	31 (34.8)	39 (41.9)	0.33
NT-proBNP, ng/L	2614 (973–5092)	2338 (1176–5043)	0.96
NT-proBNP elevation	91 (95.8)	91 (95.8)	>0.99
ECG characteristics			
Abnormal ECG	84 (87.5)	89 (92.7)	0.23
QRS abnormality	57 (59.4)	50 (52.1)	0.31
ST-T abnormality	28 (29.2)	41 (42.7)	0.05
Sustained ventricular tachycardia	1 (1.0)	1 (1.0)	>0.99
Non-sustained ventricular arrhythmia	43 (44.8)	34 (35.4)	0.19
AF	12 (12.5)	15 (15.6)	0.53
LBBB	24 (25.0)	18 (18.8)	0.30
UCG characteristics			
LVEDD, mm	66 (61–75)	66 (59–72)	0.50
LVESD, mm	58 (51–64)	57 (51–63)	0.62
LVFS, %	14 (12–18)	14 (11–17)	0.39
LVEF, %	29 (24–35)	29 (23–33)	0.76
IVS, mm	7 (7–9)	8 (7–9)	0.49
LVPW, mm	8 (7–9)	8 (7–9)	0.40
LAD, mm	45 (40–50)	45 (41–50)	0.87
RVD, mm	25 (21–30)	26 (21–31)	0.51
LA enlargement	88 (91.7)	91 (95.8)	0.24
Moderate or severe LV diastolic dysfunction	66 (78.6)	56 (76.7)	0.78
Moderate or severe MVR	36 (37.5)	30 (31.3)	0.36
CMR characteristics			
LGE *	38 (65.5)	24 (66.7)	0.91
Baseline medications			
ACEI/ARB/ARNI	79 (82.3)	89 (92.7)	0.03
Beta blocker	87 (90.6)	92 (95.8)	0.15
MRA	70 (51.9)	88 (91.7)	0.001
SGLT2i	9 (9.4)	8 (8.3)	0.80
Diuretics	66 (68.8)	74 (77.1)	0.19
Antiplatelet drugs	21 (21.9)	33 (34.4)	0.05
Anticoagulant drugs	25 (26.0)	18 (18.8)	0.23
CRT	7 (7.3)	8 (8.3)	0.79
ICD (or CRT-D)	8 (8.3)	10 (10.4)	0.62
PM (or CRT-P)	4 (4.2)	5 (5.2)	>0.99

Values are given as mean ± standard deviation, median (25th–75th percentiles), or number (%). *p* values were calculated by Student’s *t*-test (for normally distributed data) or Mann–Whitney U tests (for non-normally distributed data) for quantitative variables and chi-square test or Fisher’s exact test for qualitative variables, when appropriate. * Enhanced CMR was performed in 58 dLVNC patients and 36 DCM patients. Abbreviations: dLVNC, dilated left ventricular non-compaction; DCM, dilated cardiomyopathy; NYHA, New York Heart Association; BMI, body mass index; HGB, hemoglobin; ALT, alanine transaminase; Scr, serum creatine; FBG, fasting blood glucose; TC, total cholesterol; TGs, triglycerides; HDL-C, high-density lipoprotein cholesterol; LDL-C, low-density lipoprotein cholesterol; cTnI, cardiac troponin I; NT-proBNP, N-terminal pro-B-type natriuretic peptide; ECG, electrocardiogram; AF, atrial fibrillation; LBBB, left bundle branch block; UCG, echocardiography; LVEDD, left ventricular end-diastolic diameter; LVESD, left ventricular end-systolic diameter; LVFS, left ventricular fraction of shortening; LVEF, left ventricular ejection fraction; IVS, interventricular septum; LVPW, left ventricular posterior wall; LAD, left atrial anteroposterior diameter; RVD, right ventricular anteroposterior diameter; LA, left atrium; LV, left ventricular; MVR, mitral regurgitation; CMR, cardiac magnetic resonance; LGE, late gadolinium enhancement; ACEI, angiotensin converting enzyme inhibitor; ARB, angiotensin II receptor antagonist; ARNI, angiotensin receptor neprilysin inhibitor; MRA, mineralocorticoid receptor antagonist; SGLT2i, sodium-glucose cotransporter 2 inhibitor; CRT, cardiac resynchronization therapy; ICD, implantable cardioverter–defibrillator; CRT-D, cardiac resynchronization therapy defibrillator; PM, pacemaker; CRT-P, cardiac resynchronization therapy pacemaker.

**Table 2:** Clinical outcomes of dLVNC and DCM patients.

Outcomes	dLVNC (*n* = 96)	DCM (*n* = 96)	*p* Value
Follow-up time, years	5.46 (2.14–8.62)	5.26 (2.47–8.50)	0.89
Primary outcomes			
MACE	59 (61.5)	59 (61.5)	>0.99
Cardiovascular death or heart transplantation	16 (16.7)	23 (24.0)	0.21
Cardiovascular death	15 (15.6)	18 (18.8)	-
Heart transplantation	1 (1.0)	5 (5.2)	-
Hospitalization for HF or CRT implantation	48 (50.0)	57 (59.4)	0.19
Hospitalization for HF	47 (49.0)	56 (58.3)	-
CRT implantation	2 (2.1)	4 (4.2)	-
Severe arrhythmia events	4 (4.2)	3 (3.1)	0.70
Ventricular fibrillation	2 (2.1)	2 (2.1)	-
Sustained ventricular tachycardia	1 (1.0)	0 (0)	-
Appropriate ICD discharge	1 (1.0)	0 (0)	-
Systemic embolism	8 (8.3)	4 (4.2)	0.23
Ischemic stroke	7 (7.3)	3 (3.1)	-
Peripheral artery embolism	2 (2.1)	1 (2.6)	-
Secondary outcomes			
All-cause death	17 (17.7)	18 (18.8)	0.85
Arrhythmia events	26 (27.1)	22 (22.9)	0.51
AF	8 (8.3)	8 (8.3)	-
LBBB	2 (2.1)	2 (2.1)	-
Ventricular arrhythmia	9 (9.4)	12 (12.5)	-
PM implantation	1 (1.0)	2 (2.1)	-
ICD implantation	4 (4.2)	7 (7.3)	-
Other arrhythmia events	8 (8.3)	6 (6.3)	-

Values are given as numbers (%). *p* values were calculated by the chi-square test or Fisher’s exact test for qualitative variables, when appropriate. Abbreviations: dLVNC, dilated left ventricular non-compaction; DCM, dilated cardiomyopathy; MACE, major adverse cardiovascular event; HF, heart failure; CRT, cardiac resynchronization therapy; ICD, implantable cardioverter–defibrillator; AF, atrial fibrillation; LBBB, left bundle branch block; PM, pacemaker.

**Table 3:** Effects of LVEF and LGE on the risk of MACEs.

Risk Factor	HR (95% CI)	*p* Value
dLVNC		
LVEF ≤ 30%	1.34 (0.78–2.32)	0.29
LGE *	3.13 (1.38–7.07)	0.006
DCM		
LVEF ≤ 30%	1.39 (0.82–2.34)	0.22
LGE	1.93 (0.64–5.83)	0.24

HR (95% CI) and *p* values were calculated using the univariate Cox proportional-hazards regression model. * Enhanced CMR was performed in 58 dLVNC patients and 36 DCM patients. Abbreviations: LVEF, left ventricular ejection fraction; LGE, late gadolinium enhancement; MACEs, major adverse cardiovascular events; HR, hazard ratio; CI, confidence interval; dLVNC, dilated left ventricular non-compaction; DCM, dilated cardiomyopathy.

## Data Availability

The original contributions presented in this study are included in the article. Further inquiries can be directed to the corresponding authors.

## Abbreviations

The following abbreviations are used in this manuscript:

LVNC	Left ventricular non-compaction
DCM	Dilated cardiomyopathy
dLVNC	Dilated left ventricular non-compaction
LVEF	Left ventricular ejection fraction
LVEDD	Left ventricular end-diastolic diameter
PSM	Propensity score matching
HF	Heart failure
MACEs	Major adverse cardiovascular events
LGE	Late gadolinium enhancement
CMR	Cardiac magnetic resonance
ESC	European Society of Cardiology
ICD	Implantable cardioverter–defibrillator
PUMCH	Peking Union Medical College Hospital
NC/C	Ratio of noncompacted to compacted myocardial layer thickness
ECG	Electrocardiography
UCG	Echocardiography
BMI	Body mass index
NYHA	New York Heart Association
cTnI	Cardiac troponin I
NT-proBNP	N-terminal pro-B-type natriuretic peptide
IVS	Interventricular septal thickness
LVPW	Left ventricular posterior wall thickness
RVD	Right ventricular anteroposterior diameter
LAD	Left atrial anteroposterior diameter
LA	Left atrium
CRT	Cardiac resynchronization therapy
HR	Hazard ratio
CI	Confidence interval
LVESD	Left ventricular end-systolic diameter
LVFS	Left ventricular fraction of shortening
HGB	Hemoglobin
ALT	Alanine transaminase
Scr	Serum creatine
FBG	Fasting blood glucose
TC	Total cholesterol
TG	Triglycerides
HDL-C	High-density lipoprotein cholesterol
LDL-C	Low-density lipoprotein cholesterol
AF	Atrial fibrillation
LBBB	Left bundle branch block
LV	Left ventricular
MVR	Mitral regurgitation
ACEI	Angiotensin-converting enzyme inhibitor
ARB	Angiotensin II receptor antagonist
ARNI	Angiotensin receptor–neprilysin inhibitor
MRA	Mineralocorticoid receptor antagonist
SGLT2i	Sodium-glucose cotransporter 2 inhibitor
CRT-D	Cardiac resynchronization therapy defibrillator
PM	Pacemaker
CRT-P	Cardiac resynchronization therapy pacemaker
